# Autologous adipose tissue transfer in progressive hemifacial atrophy: From simple volume to regenerative cell therapy

**DOI:** 10.1016/j.jpra.2025.05.003

**Published:** 2025-05-11

**Authors:** M.L. Foba, V. Mégevand, M. Scampa, E.H.D. Teuw, P. Quinodoz, A.-A. Sankale, D.F. Kalbermatten, D. André-Lévigne

**Affiliations:** a Department of Plastic, Reconstructive and Aesthetic Surgery, CHNU de Fann, 15186 Dakar Fann, Senegal; b Department of Plastic, Reconstructive and Aesthetic Surgery, Geneva University Hospitals, HUG, 1205 Geneva, Switzerland; c Hôpital de la Tour, Geneva, 1217 Meyrin, Switzerland

**Keywords:** Progressive hemifacial atrophy, Scleroderma, Parry-Romberg, Lipofilling, Autologous fat transfer, Case series

## Abstract

**Background:**

Progressive hemifacial atrophy (PHA), including en-coup-de-sabre morphea and Parry-Romberg syndrome, is a rare condition characterized by unilateral atrophy of facial tissues. The etiology of PHA remains unclear, though it is generally considered to be an autoimmune disease. Current treatment approaches typically involve systemic immunosuppression to stabilize the disease, followed by reconstructive surgery to restore facial symmetry, ranging from complex flap reconstruction to autologous fat transfer (AFT) and allogenic fillers. Recent evidence supports AFT not only as a volumetric filler but also for its immunomodulatory and angiogenic properties, making it a promising supplement or even alternative to systemic immunosuppressive therapy.

**Methods:**

We present four cases of PHA treated with AFT in Dakar, Senegal, and Geneva, Switzerland. A comprehensive review of evidence supporting AFT as a cellular therapy in patients with PHA was performed discussing its potential as an effective stand-alone therapeutic option.

**Results:**

There is growing evidence that AFT has regenerative effects in fibrotic autoimmune disease, including scleroderma and PHA. This is in line with our results showing not only improved facial contours and a restoration of symmetrical fullness but also improved overall tissue quality after AFT.

**Conclusion:**

We advocate AFT to be a safe and reliable therapy in PHA, offering not only a substitute to the lost tissue but also local immunomodulatory benefits useful for tissue regeneration. Besides the benefits of combining local immunomodulatory cell therapy with the reconstructive volume restoration, this technique offers a low risk profile, cost-effectiveness and excellent accessibility, particularly relevant in low-income settings.

## Introduction

Progressive hemifacial atrophy (PHA) is a group of rare conditions including en-coup-de-sabre morphea and Parry-Romberg syndrome. It is characterized by unilateral facial atrophy of the skin, subcutaneous tissue, muscles, and sometimes osseocartilaginous structures. Parry-Romberg syndrome (PRS) can be associated with various systemic manifestations, including neurological, ophthalmological, and maxillofacial conditions.^1^ En-coup-de-sabre morphea is usually considered to be more localized and self-limiting disease than PRS generally and has a linear appearance resembling a scar from a sword strike.^2^ Several case series report overlaps of around 40 % between the two diagnoses.^3^^,^^4^ It is hence conceivable that both PRS and en-coup-de-sabre morphea are two distinctive types of localized scleroderma sharing similar pathogenesis.^2^

PHA is considered a sporadic condition more common in females with no ethnic or geographic predilection.^5^^,^^6^ The incidence of PRS has been reported to be 0.3 to 2.5 cases per 100′000 population per year and that of morphea en coup de sabre to be 2.7 per 100′000 per year.^7^ According to a report by Abdelnour et al.^8^ in 2019, only four cases have been reported in Africa, in Senegal,^9^ Libya,^10^ Morocco,^11^ and Egypt,^12^ respectively. No epidemiologic data is available on the prevalence of the disease on the African continent, but prevalence is likely to be comparable to available data in the industrialized world.

The exact cause of PHA is not known, but it is generally accepted to be an autoimmune disease. Diagnosis usually relies on clinical manifestations and radiological findings as well as autoimmune blood markers. PHA can lead to significant disfigurement involving psychosocial impairments and represents a real challenge for plastic surgeons.

Treatment modalities generally include systemic immunosuppression to stabilize the disease, followed by surgical procedures, often necessary to restore facial symmetry. There is no consensus on which surgical technique is best, especially in severe cases. Autologous fat transfer (AFT) is an emerging technique as it allows for effective long-term volume reconstruction and potentially has immunomodulatory and angiogenic effects, possibly slowing down or halting the progression of the disease.^13^

We advocate that AFT is an effective alternative and a potential stand-alone treatment of localized PHA in both high and low-income countries. We here present the cases of three patients diagnosed with PHA in their childhood who have benefitted from AFT in Dakar, Senegal during humanitarian missions, or in Geneva, Switzerland, following the STROBE (Strengthening the reporting of observational studies in epidemiology) guidelines. A narrative review was performed to discuss currently available data on the regenerative potential of AFT in fibrotic autoimmune disease in general and specifically in PHA.

## Case 1

**Figure 1:**
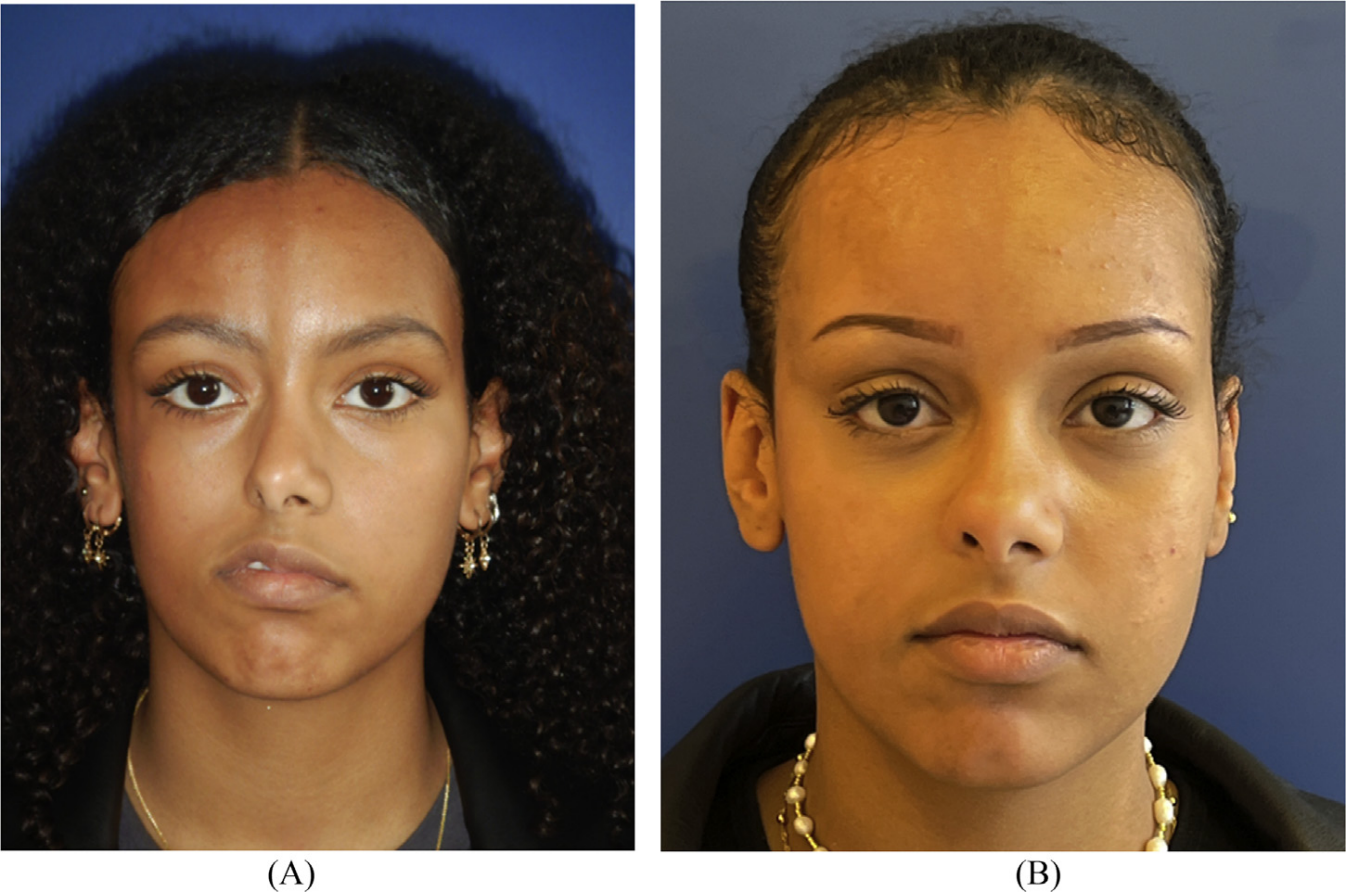
Frontal view of patient 1 before (A) and after (B) treatment with autologous fat grafting and right ala reconstruction using a retroauricular cartilage alar batten graft.

The first case is a 21-year-old female patient whose symptoms started at the age of three and treated with methotrexate (Methotrexate) and mycophenolate with poor improvement of her condition. Physical examination revealed facial asymmetry with right hemifacial atrophy and nose tip deviation towards the right side due to right alar cartilage hypotrophy (Figure 1).

A CT scan of the face revealed an atrophy of the right hemifacial subcutaneous tissues as well as an atrophy of the right alar cartilage and a septal deviation.

She underwent 3 consecutive sessions of AFT (65cc, 70cc and 40cc respectively) at the Geneva University Hospitals in Switzerland using a Body-Jet® (Human Med AG, Germany) device and a 2.5 mm liposuction cannula, with interval periods of 6 and 12 months between each procedure. Lipofilling was performed in a multidirectional manner using a 1.2 mm Coleman cannula. Injection was done in the subperiosteal plane on the zygomatic arch and the body of maxilla, insisting particularly in the region of the nasal notch to give projection to the alar crease. Subcutaneous injection was performed in the scalp and the frontal, periorbital and mental region, the right upper lip and the left alar of the nose and the tip of the nose. She also underwent a right alar reconstruction using a retro-auricular cartilage alar batten graft.

**Figure 2:**
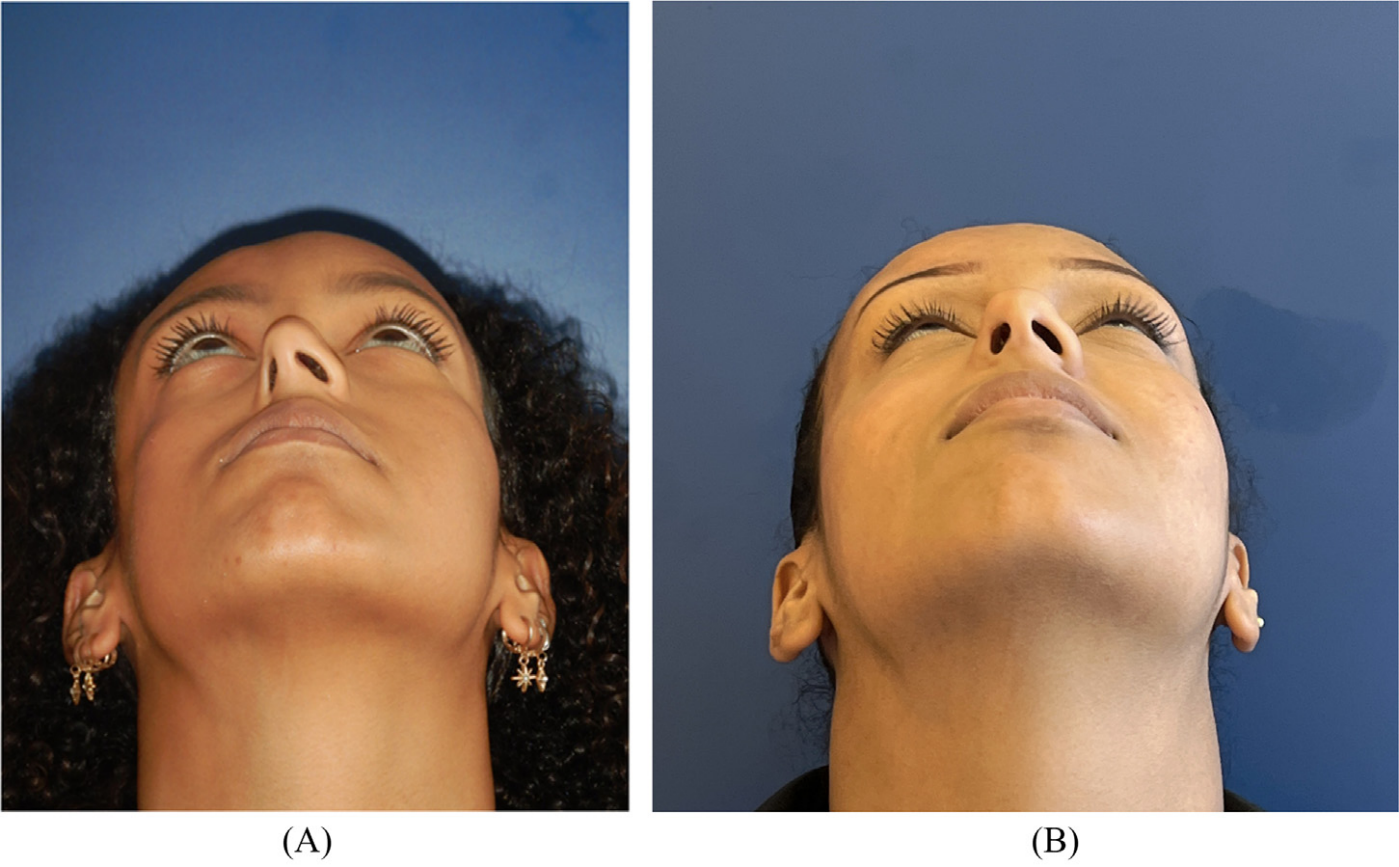
Basal view of patient 1 before (A) and after (B) treatment with autologous fat grafting and right ala reconstruction using a retroauricular cartilage alar batten graft.

**Figure 3:**
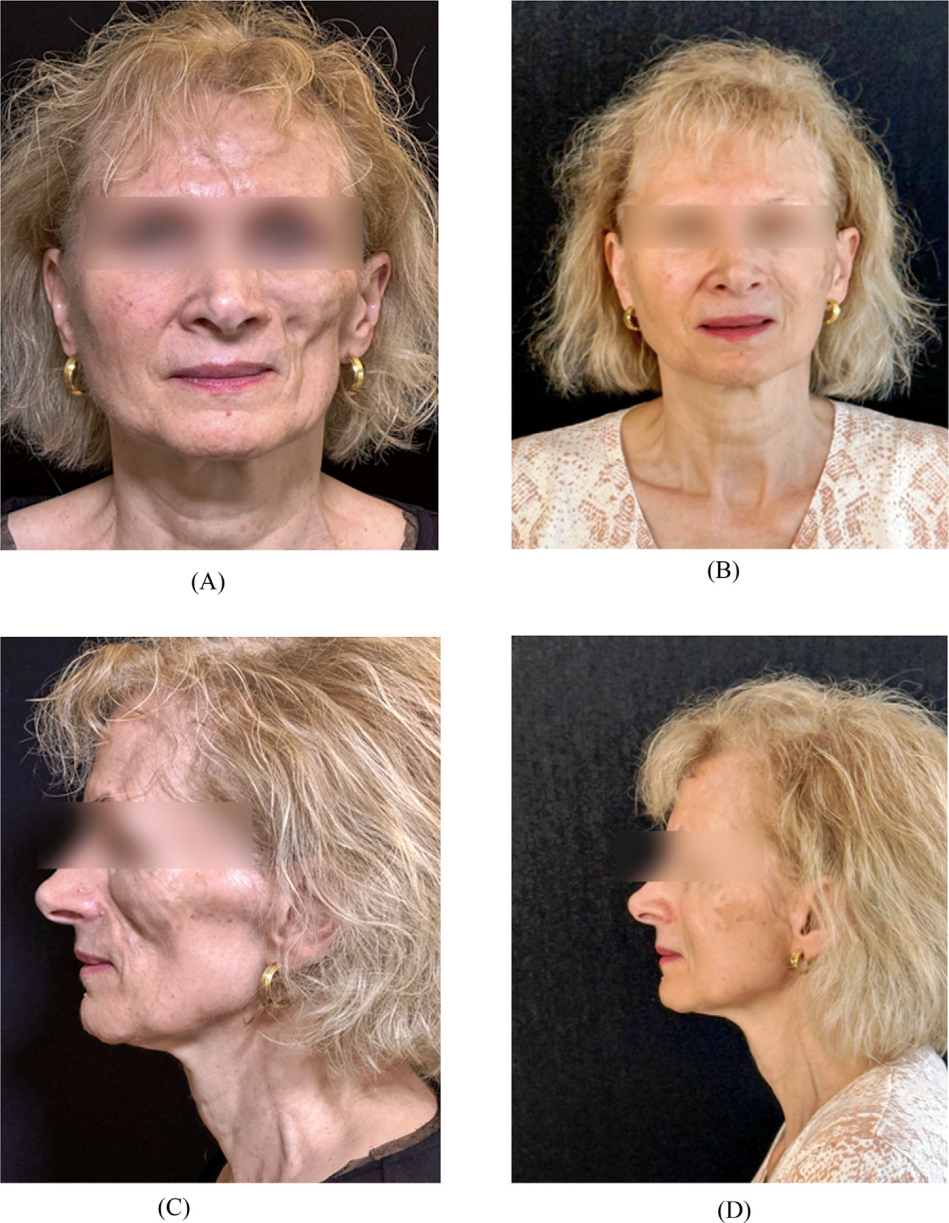
Frontal and left profile views of patient 2 before (A, C) and after (B, D) treatment with autologous fat grafting.

Three months after the final procedure, both the facial asymmetry and the right alar atrophy have significantly improved and the right hemiface is now well defined (Figure 3). Note that significant tip rotation was achieved with subperiosteal lipofilling next to the pyriform aperture as well as subcutaneous injection of the alar and tip of the nose, improving projection of the alar insertion in the cheek (Figure 2). No postoperative complications were observed.

## Case 2

The second case is a 68-year-old female patient whose symptoms appeared at the age of seventeen and who has been treated for a PRS with left hemifacial atrophy, benefitting from two consecutive sessions of AFT, respectively 30cc and 19cc, in a private center in Geneva. Liposuction was performed using a 2.5 mm cannula. Autologous fat was injected in the supra-orbital rim, in the retro-bulbar area, in the zygomatic and temporal areas, in the nasolabial fold and in the pre-tragal area. After completion of both sessions, we observed significant improvement of the left hemifacial atrophy with a harmonious delineation of the facial structures notably at the cheek bones (Figure 3). No postoperative complications were observed.

## Case 3

The third patient is a 26-year-old woman, presenting symptoms that began at the age of 19 years, marked by the progressive onset of unilateral trismus on the right side, preventing mouth opening and hindering food intake, which spontaneously improved.

This was followed by facial asymmetry and progressive atrophy of the right hemiface, with a deviation of the mouth towards the affected side without associated motor deficit.

**Figure 4:**
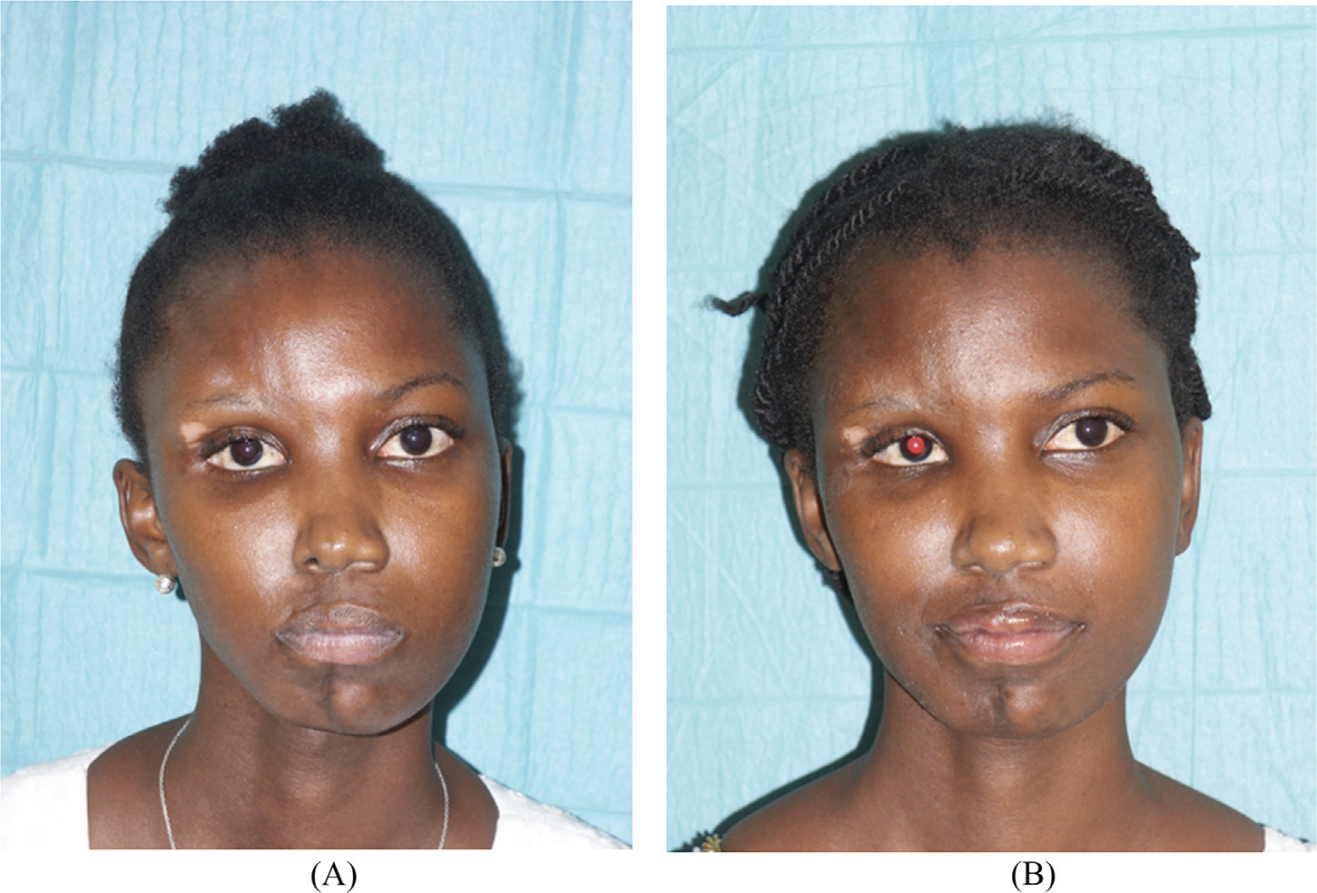
Frontal view of patient 3 before (A) and after (B) treatment with autologous fat grafting.

She received botulinum toxin injections without significant improvement. Clinical examination revealed facial asymmetry with right hemifacial atrophy, a depression line starting from the forehead, running along the right lateral edge of the nose, and ending at the upper and lower right lips and chin (Figure 4).

Electromyography (EMG), electroneuromyography (ENMG), and anti-nuclear antibody tests returned normal. A brain MRI showed normal cerebral parenchyma. A CT scan of the sinuses and face revealed left masseter muscle hypertrophy, intact temporomandibular joints, and bilateral anterior ethmoidal sinusitis. Ultimately, a facial MRI revealed radiological findings compatible with the initially clinically diagnosed PRS including right hemifacial atrophy, particularly affecting the superficial planes. She underwent 2 sessions (50cc and 40cc) of facial AFT using standard mural aspiration connected to Redon bottle through a 2.5 mm liposuction cannula for collection of fat at the plastic surgery department of the University of Dakar, Senegal. Lipofilling was performed in a multidirectional manner using a 1.2 mm Coleman cannula. Injection was done in the subperiosteal plane on the zygomatic arch and the body of maxilla, insisting particularly in the region of the nasal notch to give projection to the alar crease. Subcutaneous injection was performed in the scalp and the frontal and periorbital region.

After an 8-month follow-up, the depression line on the forehead disappeared, and AFT allowed to improve facial contour and restore symmetrical fullness of soft tissue in the frontal and midface area (Figure 4). No postoperative complications were observed.

## Case 4

**Figure 5:**
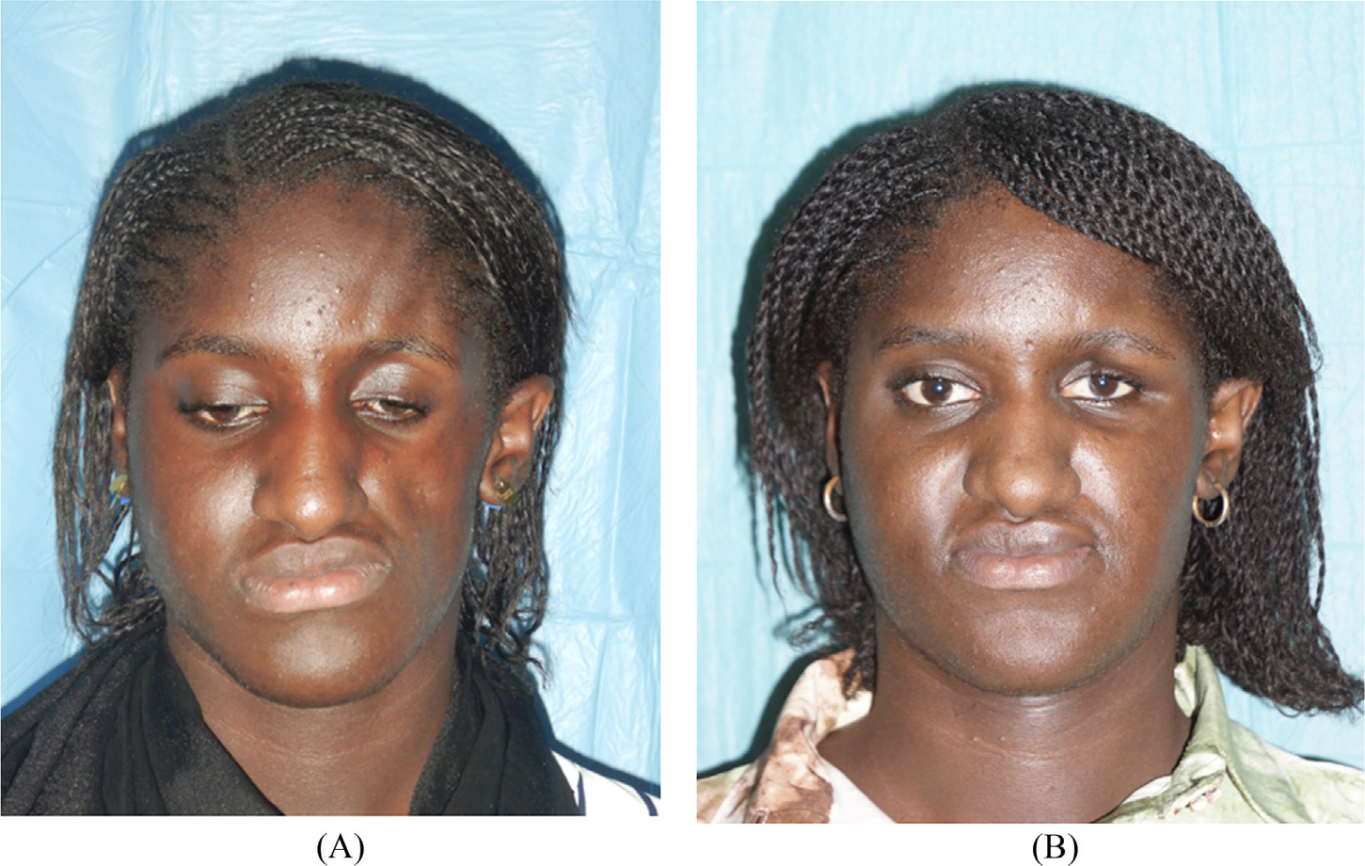
Frontal view of patient 4 before (A) and after second (B) treatment with autologous fat grafting.

The fourth case is a 17-year-old female patient with no significant medical history, whose symptoms began around the age of 7 years, marked by the progressive onset of facial asymmetry with atrophy of the left hemiface and deviation of the mouth towards the affected side without associated motor deficit. Physical examination revealed facial asymmetry with left hemifacial atrophy and a depression line starting from the forehead, running along the left lateral edge of the nose, and ending at the upper and lower left lips and chin (Figure 5).

A CT scan of the facial bones showed no bony anomaly. She underwent 2 sessions of AFT (2 × 100cc) using standard mural aspiration connected to Redon bottle through a 2.5 mm liposuction cannula for collection of fat at the plastic surgery department of the University of Dakar, Senegal. Lipofilling was performed in a multidirectional manner using a 1.2 mm Coleman cannula. Injection was done in the subperiosteal plane on the zygomatic arch and the body of maxilla, insisting particularly in the region of the nasal notch to give projection to the alar crease. Subcutaneous injection was performed in the scalp and the frontal, periorbital and mental region.

After a 20-month follow-up, the depression line resembling a sabre cut on the forehead regressed, and we could observe a more harmonious delineation of the right hemiface, notably at the cheek bones. The previous depression lines were now filled with fat and the symmetry of the face was partially restored (Figure 5). No postoperative complications were observed.

## Discussion

Evidence of PHA dates to ancient Egypt, with mummies showing craniofacial dysmorphism compatible with PHA^2^ but information on its pathophysiology is based on small case series or case reports, reliable data still being lacking. With the current body of evidence, an acquired etiology of PHA is widely accepted, as opposed to congenital hemifacial atrophy also known as hemifacial microtia, but familial cases have also been described, autosomal dominant transmission with incomplete penetrance being suggested, however not supported to date.^14^ Etiologies such as vascular, post-traumatic, infectious as well as hyperactivity of the sympathetic trunk have been suggested but the main theory is that PHA is an autoimmune disease.^15^ In fact, other autoimmune diseases such as inflammatory bowel disease (5 %), rheumatoid arthritis (4 %), systemic lupus erythematosus (2 %) and ankylosing spondylitis (2 %) have been observed in patients with PHA.^15^^,^^16^ Antinuclear antibodies are frequently encountered, although serologies are of limited utility for diagnosis as they lack sensitivity.^3^ In fact, antinuclear antibodies are reported to be present in only 25 to 52 % of patients with PHA.^2^ In our patients, antinuclear antibodies tests were found positive in patients 1 and 2 and negative in patients 3 and 4.

Histological findings include dermal fibrosis, loss of subcutaneous fat, and inflammatory infiltrates composed of lymphocytes and plasma cells.^17^ Epidermal skin involvement is minimal, but the tongue, gums, teeth, and palate may also be affected.^18^

Immunosuppressant therapy such as methotrexate (Methotrexate) and corticosteroids are generally used to halt the progression of PHA,^19^ methotrexate (Methotrexate) being the standard therapy for active disease.^20^ It is often combined with a regimen of prednisone for two months, followed by a gradual tapering during the third month or high-dose pulse intravenous methylprednisolone for 3 days monthly during six months, to achieve anti-inflammatory effects while minimizing side effects.^19^ Finally, antimalarial agents^2^ and Ultraviolet A (UVA) therapy have also shown some efficacy in limited cases of both scleroderma^21^ and PRS.^22^ Data on the efficacy of systemic treatments is still very limited with a systemic literature review conducted in 2021 finding data on only 69 patients in total and methotrexate (Methotrexate) being the most reported treatment.^20^

Surgery is traditionally considered for reconstruction of the lost tissue once the disease has been pharmacologically stabilized. Today, the general goal of surgery in patients with PHA is to minimize the psychosocial impact of the disease by correcting deformities and often requires multiple procedures to achieve lasting results.^23^ A recent review article including a total of 824 PHA patients found that 61 % were managed by microvascular free flaps, 37 % by AFT and 2 % by pedicled flaps. They concluded that today serial fat grafting is the primary modality used for patients with mild soft-tissue atrophy, whereas microvascular free flaps widely remain the treatment of choice for reconstruction of large-volume defects.^23^ In severe cases, the underlying osseous framework is usually affected and historically the transfer of vascularized tissue has been considered more effective. Local flaps such as the galea flap are suggested to provide volume and tissue softness.^24^ As microvascular free flaps can provide large quantities of soft tissue, they have been advocated in advanced stages of the disease. However, they come with numerous limitations as they are relatively invasive methods, require multiple steps for an optimal outcome, sometimes imply unacceptable scarring and require advanced surgical expertise.^25^ Recent literature focuses on the role of graft supplementation to improve fat graft survival after AFT, and there is growing evidence that early AFT may help curb disease progression.^23^

In our case series we demonstrate that good cosmetic results can be achieved with AFT only, including in severe cases. We advocate that AFT, if applied in the right anatomical layer, allows for excellent results, including the correction of loss of bony projection. Autologous fat not only provides a malleable filler allowing for large soft tissue reconstructions, but it also holds regenerative potential due to its high concentration of mesenchymal stem cells. In fact, AFT has been shown to effectively improve functional and esthetic outcomes in patients with systemic scleroderma with orofacial involvement.^26^ Of note, AFT has recently been suggested as an alternative to the medical treatment altogether and hence potentially as a first line therapy of craniofacial localized scleroderma.^13^

There is still limited data available specifically on PHA but data on similar fibrotic autoimmune disorders point toward a significant potential of autologous fat transfer as an immunomodulatory cell therapy. In fact, numerous studies have demonstrated the immunomodulatory role of adipose tissue.^27,^^28^ Human adipose tissue is rich in mesenchymal stem cells from and holds a high regenerative potential.^29^ It is obtainable in large quantities, even under local anesthesia, with negligible donor site morbidity, and ethically less controversial than embryonic stem cells.^30^ The proliferative and differentiation potential of adipose-derived mesenchymal stem cells has been largely demonstrated offering a wide range of therapeutic potential, notably in regenerative surgery.^31^ For instance, Strong et al. found that AFT subjectively and qualitatively improved perioral skin quality, facial animation, hand range of motion, and hand pain in patients with systemic scleroderma.^32^ Taken together, AFT represents a cost-effective, accessible and safe method to restore volume, improve skin texture and to potentially stop the progression of the disease due to the ability of its adipose-derived mesenchymal stem cells (ADSCs) to secrete immunomodulator and angiogenic factors that activate tissue regeneration.^13^

**Figure 6:**
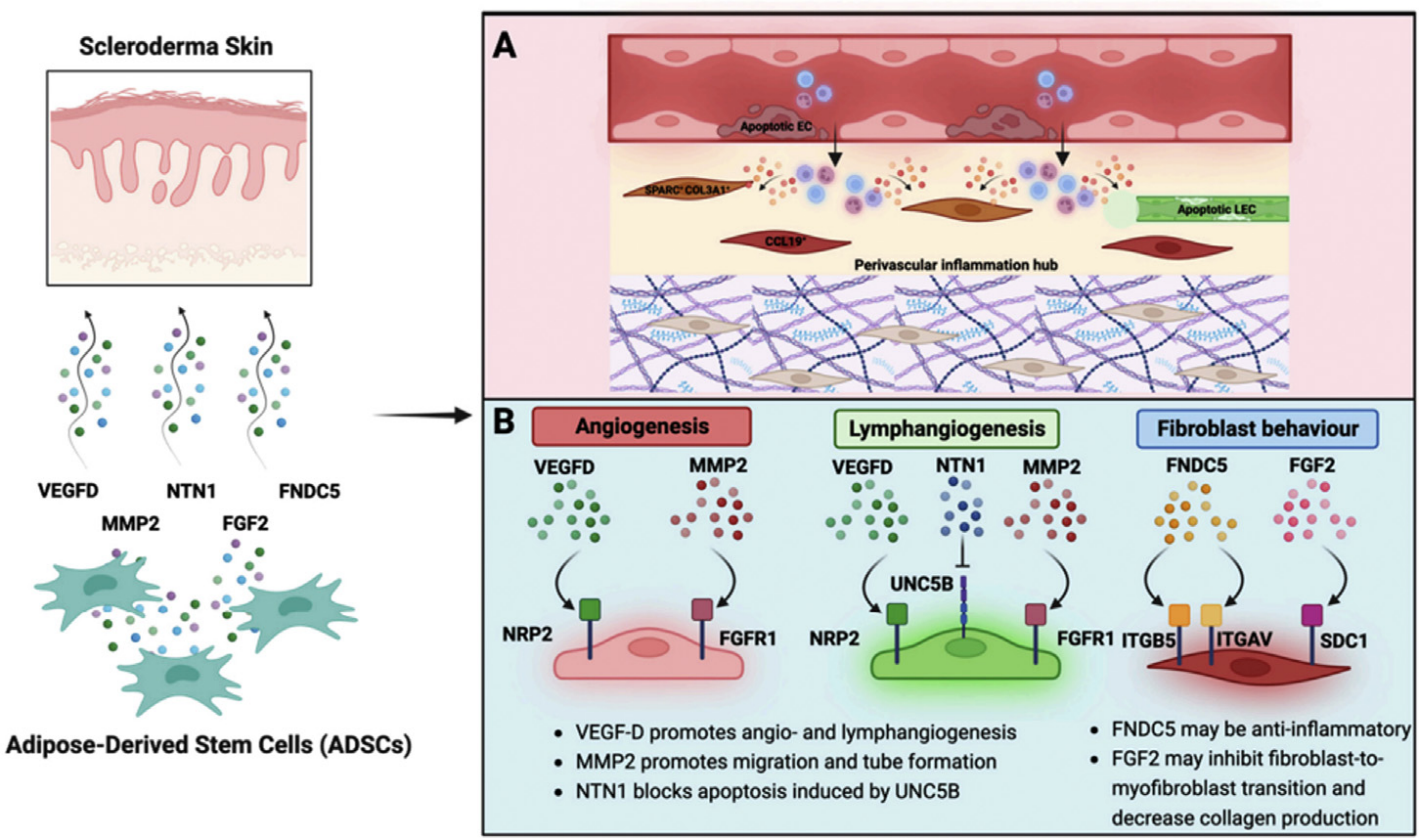
Reprint from Frommer et al.^33^: Summary of possibly therapeutic interactions of ADSCs with fibroblasts and endothelial cells from scleroderma. (A) Single-cell analyses of chronic inflammatory diseases and scleroderma identified a proinflammatory CCL19+ and a SPARC+/COL3A1+ fibroblast subset that colocalises with the vasculature, highlighting the importance of the perivascular inflammation hub for activation of fibroblasts in scleroderma. (B) Based on differences and commonalities in cell-to-cell receptor–ligand interactions of ADSCs with fibroblasts and endothelial cells from healthy and scleroderma skin, VEGFD, MMP2, NTN1, FNDC5, and FGF2 were identified as possible anti-fibrotic effector molecules.

ADSCs exhibit a unique capacity to interact with various cell types within the skin, including fibroblasts and endothelial cells, which are crucial in the pathophysiology of scleroderma. Frommer et al.^33^ simulated the effect of ADSCs on scleroderma skin using RNA sequencing datasets and found that most interactions were based on extracellular matrix proteins. They identified NTN1, VEGFD, MMP2, FGF2, and FNDC5 as potential players in how the ADSCs secretome may disrupt vascular and perivascular inflammation hubs in scleroderma and thereby promote angiogenesis and lymphangiogenesis. In an in vivo model of scleroderma in mice, Jiang et al.^34^ studied the effect of standard lipofilling, ADSCs-enriched AFT and injection of stromal vascular fraction (SVF) gel on dermal sclerosis and inflammation. They could demonstrate significantly lower levels of apoptotic cells and inflammation, reversed skin sclerosis, most significantly in ADSCs-enriched lipofilling and in SVF gel injections. They concluded that ADSCs can suppress dermal inflammation by inhibiting activated macrophages, thereby mitigating the inflammatory response associated with scleroderma. As scleroderma is characterized by an imbalance between pro-inflammatory and anti-inflammatory mediators it is conceivable that immunomodulatory mechanisms are at the core of the effect of ADSCs on scleroderma (Figure 6).^33^

There is also some clinical evidence supporting the efficacy of ADSCs in reversing fibrosis in systemic sclerosis. In a cohort of 62 patients with systemic scleroderma treated by orofacial lipofilling, Almadori et al.^35^ studied the effect of orofacial AFT in patients with systemic sclerosis. They evaluated clinical outcomes and studied the effects of ADSCs in co-culture models. They found that AFT significantly improved functional and psychological outcomes in patients with peri‑oral sclerosis and that AFT may reduce dermal fibrosis through the suppression of fibroblast proliferation and key regulators of fibrogenesis including TGF-β1 and CTGF. Of note, Okamura et al.^36^ found that systemically injected ADSCs attenuated skin and lung fibrosis in bleomycin (Bleomycin)-induced scleroderma in mice. They found lower levels of mRNA expression of collagen and fibrogenic cytokines, such as interleukin (IL)-6 and IL-13.

The mechanisms underlying the immunomodulatory effects of AFT are likely to be multifaceted and may not be limited to the effects of ADCSs but might also include adipocytes themselves. In a model of inguinal fat pad transplantation in bleomycin (Bleomycin) induced fibrosis in mice, Wang et al.^37^ report that adipocytes transform into a more functional and dedifferentiated state and reverse dermal fibrosis. They found that both ADSCs and dedifferentiated adipocytes promote dermal fat tissue regeneration, improve angiogenesis, suppress macrophage-mediated inflammation and myofibroblast accumulation. They observed in fact a shift from pro-inflammatory (M1) to anti-inflammatory (M2) macrophage phenotypes. Accordingly, it has been reported that intraperitoneal injection of ADSCs has an immunomodulatory effect via M2 macrophage switch in an inflammatory bowel disease model in mice.^38^

In addition to their immunomodulatory effects, ADSCs also play a role in restoring adipose tissue of the recipient site, which is often depleted in scleroderma patients. Fat grafting, hence, does not only deliver new adipocytes to the affected area but also induces regeneration of the existing fat tissues. In fact, Quan et al.^39^ found that the transplantation of ADSCs is associated with the regeneration of subcutaneous fat.

All this evidence feeds into a greater picture of the potential immunomodulatory effects of ADSCs in a great variety of autoimmune diseases where they have been shown to enhance the secretion of growth factors that facilitate tissue regeneration and repair.^40^

Simonacci et al.^41^ discuss the clinical applications of ADSCs in regenerative medicine, highlighting their ease of harvest, high yield, and multi-lineage potential. The authors emphasize that, according to European and U.S. regulations, many clinical trials involving cultured ADSCs or their use in non-homologous tissues may be considered off-label, necessitating adherence to specific regulatory guidelines for safety and efficacy.

AFT is considered very safe with clinical complications reported to be at 3.7 %, mainly irregularities and asymmetries and 0.1 % infections and 0.6 % hematoma.^42^ Very rare severe complications, such as unilateral vision loss and cerebral infarction due to fat embolism, have been reported.^42^

The main limitations of AFT is the unpredictable retention rate.^43^ A meta-analysis on the efficacy of AFT in facial reconstructive surgery found 50 % to 60 % of the injected volume was retained at 1 year.^42^ In patients with PHA, fat retention rates after AFT may be even lower. In a meta-analysis including 1,572 patients with facial deformities, the authors found that autologous fat retention rates in patients with PRS and en coup-de-sabre scleroderma was only 43 %.^44^ This difference may be caused by the abnormalities of the immune system and fibrosis in the affected population. In addition, in this group of patients, treatment with immunosuppressive agents such as glucocorticoids may reduce the number of adipose stem cells, leading to poor surgical outcomes. Hence, repeated injection sessions are usually necessary and numerous techniques have been investigated to improve graft survival, including platelet rich plasma (PRP), ex-vivo expanded ADSCs, stromal vascular fraction (SVF) and others.^45^^,^^46^

**Figure 7:**
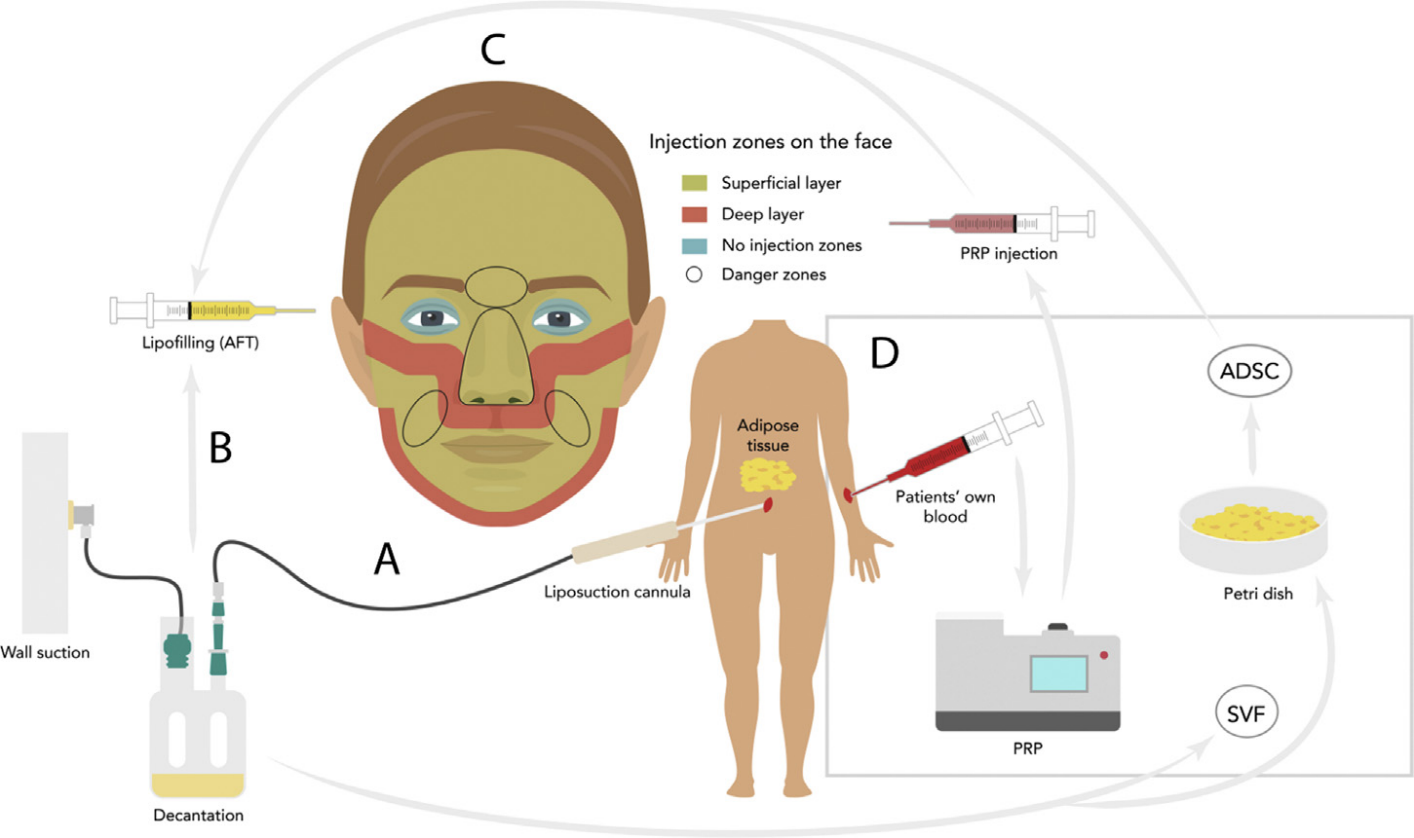
Schematic illustration of the use of AFT in hemifacial atrophy patients with standard surgical equipment and with popular optional enhancement methods: (A) A standard wall aspiration is connected to a Redon drain bottle and a 2.5 mm liposuction canula. He fat is left for decantation in the bottle. (B) After decantation, the fat is extracted from the bottle and injected into the face. (C) Schematic illustration of the injection zones of the face. (D) Illustration of popular methods to enhance the survival of AFT. PRP, platelet rich plasma; ADSC, adipose-derived stem cells; SVF, stromal vascular fraction.

**Figure 8:**
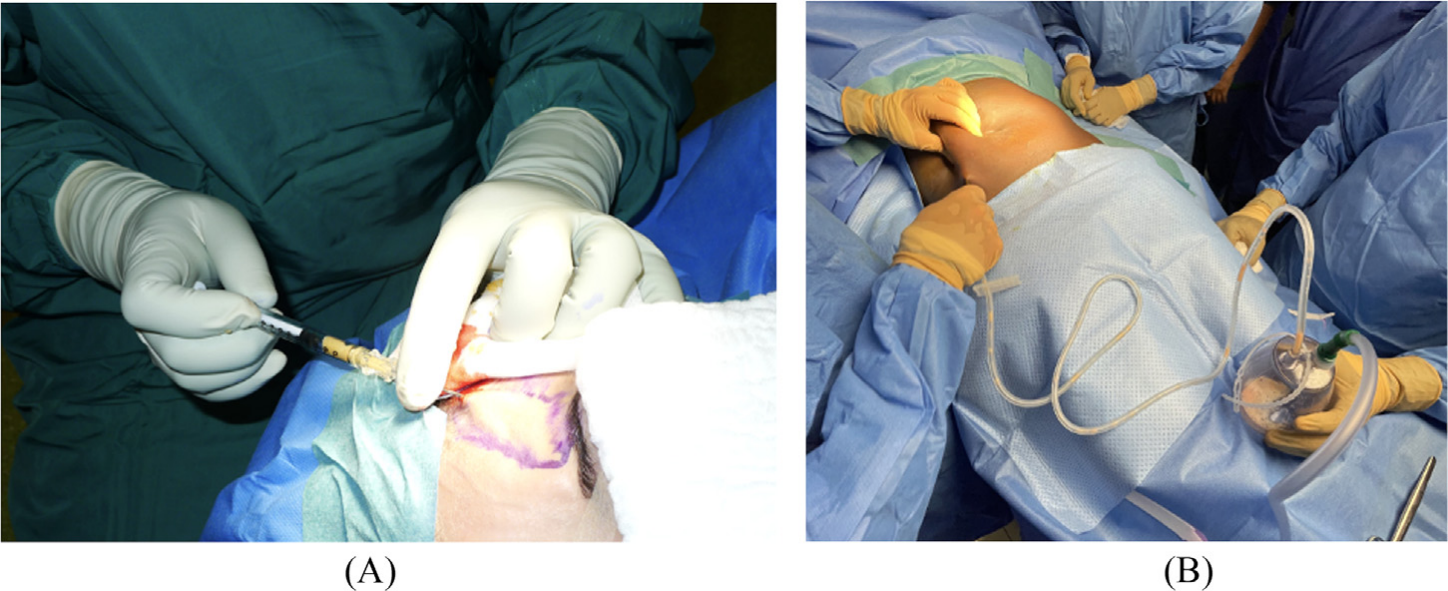
(A) Autologous fat transfer after decantation using a Coleman cannula. (B) A standard mural aspiration and a Redon surgical drain connected to a Coleman cannula used as a cost-efficient alternative for autologous fat harvesting.

Fat tissue harvest is usually performed with a sterile collector system such as LipoCollector (Human Med AG, Germany), BodyJet (Human Med AG, Germany) or MicroAire (MicroAire Surgical Instruments LLC, USA). These systems are designed to facilitate fat harvest and reduce donner site bruising. After decantation or centrifugation, the fat can be ether injected pure or in adjunction with survival-enhancing products. PRP, which can be easily harvested from the patient’s own blood has been reported to enhance neovascularization, adipocyte viability and AFT intake.^47^^,^^48^ SVF or expanded ADSCs can also be added to the lipoaspirate and are obtainable from the fat tissue itself. The lipofilling is performed by injected into the targeted areas in a multidirectional manner using a Coleman cannula, in the subcutaneous or subperiosteal plane, depending on the anatomical region. A schematic representation of the use of AFT and popular techniques to enhance fat graft survival are illustrated in Figure 7. These techniques require more sophisticated equipment and are not available in all settings. To overcome the issue of equipment availability when performing AFT in low-resource environments, we here show that standard mural aspiration and a Redon surgical drain connected to a Coleman cannula can be used as a cost-efficient alternative (Figure 8).

An alternative to AFT is the use of synthetic and biologic fillers. Different types have been designed and approved to augment volume in the facial area. Hyaluronic acid (HA) is frequently used in cosmetic applications to improve midface fullness and provide aesthetic improvements. Different formulations are available on the market with various density and elasticity. Usually, high density and cohesivity formula are used for deep injection such as sub-periosteal to add volume and bulk whereas less dense and cohesive formula are used more superficially to correct small contours deformity. In PHA, HA has been successfully used to augment soft tissue volume.^49^^,^^50^ Chaima et al.^50^ described two cases treated with high density HA with one case that provided durable results for up to 2 years.

An alternative to HA is the use of Poly-l-Lactic acid (PLLA), an approved semi-permanent filler used in the treatment of facial atrophy in HIV patients that stimulates collagen deposition by macrophages through an inflammatory reaction. This filler can also be used in PHA and bears the advantage of providing more durable results than hyaluronic acid reported to last up to 25 months in cosmetic surgery patients.^51^ Use of calcium hydroxyalapatite (CaHA), a synthetic filler approved for use in HIV lipo-atrophy and polyacrylamide hydrogel (PAAG) have been described as alternative fillers to correct PHA.^52^ As opposed to HA, no dissolving substance exists for PLLA, CaHA and PAAG and their injection should be considered carefully as overcorrection and infectious complications are difficult to manage.

Drawbacks of synthetic fillers in general are potential granulomatous reaction to the fillers, long term higher costs than AFT if reinjection is required, infection risk and non-durable results. However, some authors defend the use of synthetic fillers instead of AFT arguing that PHA comes from a fat metabolism disturbance and therefore synthetic fillers would be less subject to resorption than AFT but no comparative data is available to substantiate this theory.^49^ On the contrary, it is conceivable that AFT has a beneficial effect on the local fat tissue, as discussed earlier. PHA reactivation after filler injection was suspected in one study from Fan et al.,^53^ however no information on the filler type was provided.

In severe PHA, alloplastic prosthetics have been used instead of bone grafting to augment volume while avoiding multiple sessions of AFT or injectable fillers.^54^ Use of silicone, titanium mesh, polyethylene and expanded polytetrafluoroethylene (ePTFE) implants have been described.^55^^,^^56^ Those implants can provide sufficient volume in cases where bone asymmetry is present. Drawbacks of alloplastic prosthesis are potentially visible scars, risk of implant displacement and of infection.

Given the advantages of AFT over other surgical techniques, notably its ease of use and cost-effectiveness, we believe it is of great benefits for surgeons practicing in high and low-resource environments to use it in their lines of treatment for PHA. We also believe that AFT could become eventually a safe and reliable first line alternative to the medical treatment of craniofacial localized scleroderma, offering local immunomodulatory benefits useful for tissue regeneration without the potential side effects of a systemic immunosuppressant therapy.

## Conclusion

Little data is available on the pathophysiology and management of PHA. AFT is an easy-to-use and cost-effective therapeutic modality to correct facial deformities associated with the disease, including in severe cases as demonstrated in this case series. AFT is highly accessible, including in low-resource environments. Emerging data suggests that AFT might have a local immunomodulatory effect through its ADSCs and dedifferentiating adipocytes. The immunomodulatory effects of ADSCs in scleroderma are supported by a growing body of evidence. Their ability to modulate immune responses, suppress inflammation, and promote tissue regeneration positions them as a promising therapeutic option for managing this complex disease. ADSCs-enriched AFT potentially represents an alternative to systemic immunosuppressant therapies. In fact, AFT could eventually become a first line therapy in the management of craniofacial localized scleroderma, shifting the role of surgery from a palliative to a curative one.

## Fundings

None.

## Ethics

written consent has been obtained by all the patients for photo publication. This study is a retrospective case series, therefore no ethical approval has been requested.

## CRediT authorship contribution statement

**M.L. Foba:** Conceptualization, Writing – original draft, Methodology. **V. Mégevand:** Conceptualization, Writing – original draft, Methodology. **M. Scampa:** Writing – original draft, Methodology. **E.H.D. Teuw:** Methodology. **P. Quinodoz:** Methodology. **A.-A. Sankale:** Writing – review & editing, Supervision. **D.F. Kalbermatten:** Writing – review & editing, Supervision. **D. André-Lévigne:** Conceptualization, Writing – review & editing, Supervision.

## Conflict of interest

One of the authors is an editor for JPRAS and was not involved in the editorial review or the decision to publish this article. All remaining authors declare no conflict of interest.
